# From the Home to the Hospital

**Published:** 1887-01-22

**Authors:** 


### FROM THE HOME TO THE HOSPITAL.

The British people have the defects of their virtues. Conspicuous among their excellences are good-sense, energy, and a perseverance which almost amounts to doggedness. Whether the great Napoleon ever said they did not know when they were beaten, is, perhaps, a little doubtful; but the fact is quite true that, like their own bulldogs, they are exceedingly difficult to conquer. Their defects seem to be the necessary accompaniments of these very qualities. A certain want of imagination, of the power of abstraction, of clear and sharp definition, is a notable peculiarity among us. If a Frenchman could add to his natural clearness of vision the force and continuity of the Englishman, he would beat the islander out of the field. On the other hand, if the calm and enduring Briton could see with the eagle-like quickness of the Frenchman, and realise ideas with the same mental precision, he would have no equal for efficiency among all the peoples of the world.

These remarks are occasioned by the nature of the subject we are about to bring before the reader. We desire to see a *bridge* between the poor man's cottage and the hospital. Our sympathy with those who, often with much self-denial, supply the resources for the humane management of the sick poor is great. But though our sympathy with an over-solicited public is considerable, our tenderness towards the sick and suffering is still greater. And here is the reason why we deplore the want of a vivid and realising imagination among our people. If they could but know in their hearts and make real to themselves the most painful and distressing sufferings which poor people often endure in their removal from their homes to the hospital, the easy bridge which we have in view would speedily become an accomplished fact.

The *bridge* here spoken of is of course an efficient *ambulance system*. It is not necessary to describe at length what is meant by an ambulance system. Probably many persons are under the impression that such a system is already in existence and in full operation at all our considerable hospitals. There is a prevalent idea that the hospital system of this country has attained such a degree of perfection that nothing really necessary remains unaccomplished. Such, unhappily, is by no means the case, because the public have not supplied them with adequate means.

There is, therefore, in this country no general and regularly organised system of ambulance. Patients requiring to be removed to a hospital, no matter what their distance from it, no matter what the painful and dangerous nature of their ailment, must get there as they can. If hospital patients belonged to the middle and upper classes, they might, and no doubt would, provide for themselves the means of safe and painless transit. But, as is well known, they belong almost exclusively to the poorer sections of the community, and have absolutely no means whatever of safe and easy removal to the hospitals. The very thought of a cab to a man with a broken leg, or a woman with a painful internal disease, is almost sickening. And yet this is the easiest mode of carriage which the thousands of hospital patients have been able to command for the last hundred years, and the easiest they are able to command to-day. It is not that our people are wanting in pity; it is not that their hearts are cold. The hospital system itself is a sufficient proof of their sympathy with distress. But the richer people who support these institutions have no personal experience, as the poor have, of the three or four miles' journey to the nearest hospital in a small stiff box on wheels like the London cab. Having no personal experience, they cannot realise the suffering; and the poor themselves are so accustomed to hardships and to the present condition of matters, that they make no loud outcry, and so the thing goes on from year to year.

But that ought not to be. The object of this article is not to fill the pages of a newspaper with so much padding, but to urge earnest consideration of this long-delayed subject. The hospitals without a system of ambulance are incomplete, and incomplete in a highly important particular. They cannot do their work as perfectly as it is possible to be done, so long as patients are taken to them in a half-dying condition, as the result of their dreadful journey over the stones of the crowded streets in a wretched cab. The subject cannot be contemplated with patience, and the present state of things is only tolerated because, as we have already said, we are hardened by custom, and have no imagination.

The present writer would like to take the philanthropic public, and the non-philanthropic public also, to a railway accident eight or ten miles out of London. The most custom-hardened and stolid of the company should be put into a cart or cab—a cart, probably—in company with a child having a broken leg, the bone protruding, as it often does, through the lacerated skin. He should see the poor little fellow fainting from pain and loss of blood. He should see him restored by stimulants. He should see him jolted over ruts and stones—each jolt tearing the flesh and nerves, and causing him to scream or faint with agony—for a distance of some miles, and a prolonged period of time. Then he should see the poor child finish his journey more dead than alive from the pain; and perhaps requiring to lose his limb as the result of injuries made tenfold more intense and dangerous by this cruel method of transit to the hospital. Had there been an ambulance available, the journey would have been nothing. The child could have been put *to bed* smoothly and easily in the ambulance, and, regardless of jolt or other movement, might have *slept* the whole length of the journey.

Such are the circumstances which it is desired to make a sympathetic public realise. The Hospitals Association, Norfolk House, W.C., have resolved to take up the matter with energy, and without delay. It is proposed and hoped in due time to establish in each large centre of population an efficient ambulance system which shall be as promptly available as a fire-escape in time of need. It is hoped to convert every line of railway in the kingdom into a cheap and easy means of transit where needed, from the cottage to the ambulance, or direct to the hospital; and, by bringing the railways into permanent relationship with the ambulance system through The Hospitals Association, to put an end at once and for ever to the possibility of such painful and dangerous journeys as we have described. Will the hospitals and the public unite to add the crowning distinction of an ambulance service to the vast and beneficent system which a noble philanthropy has built up, by the creation of a bridge, whereby the sick and the suffering may reach the needed help without pain and danger?

